# 2,6-Di­bromo-4-methyl­aniline

**DOI:** 10.1107/S2414314622005776

**Published:** 2022-06-07

**Authors:** Ouarda Brihi, Meriem Medjani, Hassiba Bougueria, Amel Djedouani, Michelle Francois, Solenne Fleutot, Ali Boudjada

**Affiliations:** a Laboratoire de Cristallographie, Département de Physique, Université Frères Mentouri-Constantine 1, 25000 Constantine, Algeria; b Laboratoire de Cristallographie, Département de Physique, Université Mentouri-Constantine 1, 25000 Constantine, Algeria; c Unité de Recherche de Chimie de l’envirenement et Moléculaire Structurale (CHEMS), Département de Chimie, Faculté des Sciences Exactes, Université de Constantine 1, 25000 Constantine, Algeria; d Laboratoire de Physicochimie Analytique et de Cristallochimie de Matériaux Organo-métalique et Biomoléculaire, 25000 Constantine, Algeria; e Institut Jean Lamour UMR, 7198 Parc de Saurup CS 14234, F 54042 Nancy, France; University of Aberdeen, Scotland

**Keywords:** aniline, crystal structure, N—H⋯N hydrogen bonds

## Abstract

In the title compound, C_7_H_7_Br_2_N, the C—C—C bond angles of the benzene ring are notably distorted and two short intamolecular N—H⋯Br contacts occur. In the crystal, the mol­ecules are linked by N—H⋯N hydrogen bonds to generate *C*(2) chains propagating in the [100] direction.

## Abstract 2

The mol­ecules of the title compound are linked by weak N—H⋯N hydrogen bonds into [100] chains.

## Graphical abstract

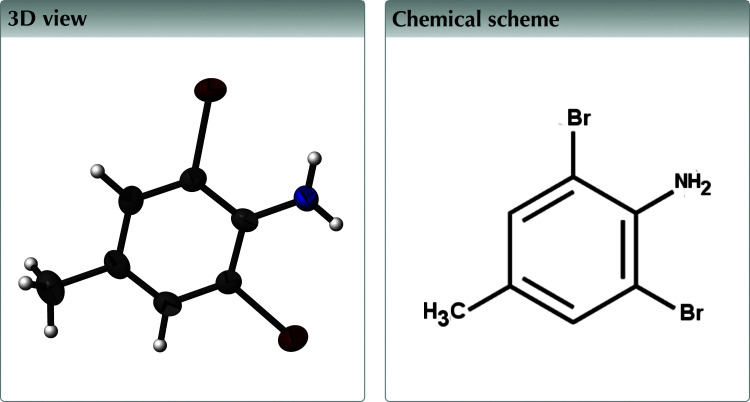

## Structure description

**Figure 1:**
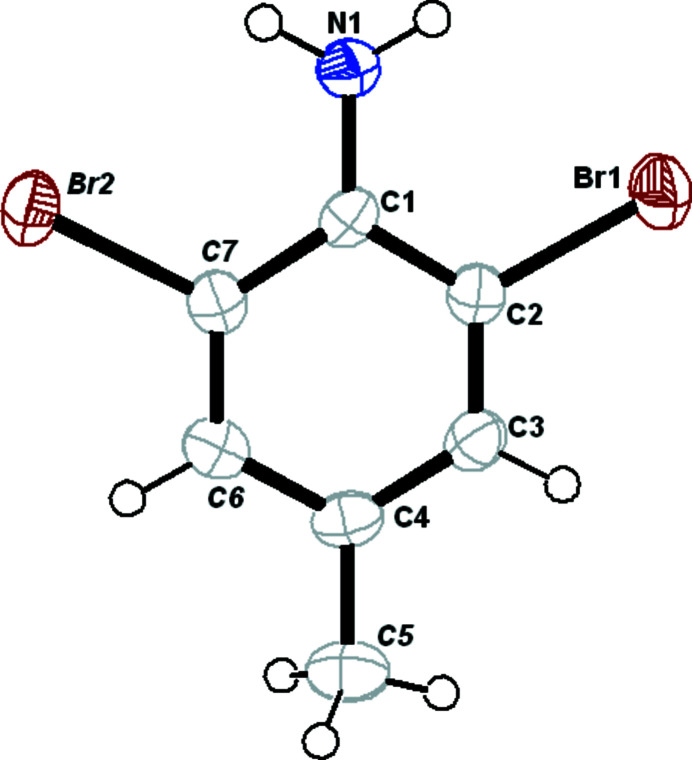
The mol­ecular structure of the title compound showing displacement ellipsoids at the 50% probability level.

The solid-state structure of the title compound, C_7_H_7_Br_2_N, was established by single-crystal X-ray diffraction analysis at 200 K and the mol­ecular structure is illustrated in Fig. 1 ▸. The bromine atoms are slightly displaced from the mean plane of C1–C4/C6/C7 benzene ring, by 0.032 (1) and 0.065 (1) Å for Br1 and Br2, respectively. This can also be qu­anti­fied by the C4—C3—C2—Br1 and C4—C6—C7—Br2 torsion angles, which are 179.7 (3) and −178.5 (3)°, respectively. The bond angles in the benzene ring are notably distorted from the ideal value of 120° with C7—C1—C2 = 115.1 (4), C1—C2—C3 = 122.8 (4) and C1—C7—C6 = 123.0 (4)°. The amine group lying between the bromine atoms results in two short intra­molecular N—H⋯Br contacts (Table 1 ▸).

**Figure 2:**
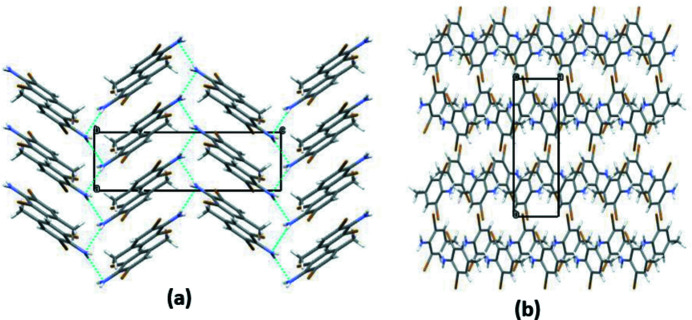
Views along the (*a*) *b* and (*b*) *c* axes of the crystal packing of the title compound with hydrogen bonds shown as dotted lines.

In the crystal, the mol­ecules are linked by weak N1—H1*B*⋯N1 hydrogen bonds (Table 1 ▸) with N⋯N = 3.120 (7) Å to generate [100] *C*(2) chains with adjacent mol­ecules related by the 2_1_ screw axis. A similar hydrogen bond was observed in di­amino­mesithylene (Brihi *et al.*, 2016 ▸). The packing is illustrated in Fig. 2 ▸, which shows the topology of the chain is a zigzag, with an angle of inclination of the benzene ring to the *a* axis of 53.73 (14)°.

## Synthesis and crystallization

The title compound is commercially available (Lancaster Synthesis). It was purified by recrystallization from a solution of 80% ethanol and 20% distilled water. The colorless single crystals obtained are in the form of needles, which grow along the *a* axis.

## Refinement

Crystal data, data collection and structure refinement details of the compound are summarized in Table 2 ▸.

## Supplementary Material

Crystal structure: contains datablock(s) I. DOI: 10.1107/S2414314622005776/hb4398sup1.cif

Structure factors: contains datablock(s) I. DOI: 10.1107/S2414314622005776/hb4398Isup2.hkl

Supporting information file. DOI: 10.1107/S2414314622005776/hb4398Isup3.cml

CCDC reference: 2175519

Additional supporting information:  crystallographic information; 3D view; checkCIF report

## Figures and Tables

**Table 1:** Hydrogen-bond geometry (Å, °)

*D*—H⋯*A*	*D*—H	H⋯*A*	*D*⋯*A*	*D*—H⋯*A*
N1—H1*A*⋯Br1	0.86	2.65	3.077 (4)	112
N1—H1*B*⋯Br2	0.86	2.64	3.072 (4)	113
N1—H1*B*⋯N1^i^	0.86	2.38	3.120 (7)	144

Symmetry code: (i) 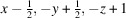 .

**Table 2:** Experimental details

Crystal data	
Chemical formula	C_7_H_7_Br_2_N
*M*_r_	264.96
Crystal system, space group	Orthorhombic, *P*2_1_2_1_2_1_
Temperature (K)	200
*a*, *b*, *c* (Å)	4.3773 (7), 13.585 (2), 14.057 (3)
*V* (Å^3^)	835.9 (2)
*Z*	4
Radiation type	Mo *K*α
μ (mm^−1^)	9.62
Crystal size (mm)	0.12 × 0.05 × 0.04

Data collection	
Diffractometer	Bruker APEXII QUAZAR CCD
Absorption correction	Multi-scan (*SADABS*; Bruker, 2016 ▸)
*T*_min_, *T*_max_	0.396, 0.746
No. of measured, independent and observed [*I* > 2σ(*I*)] reflections	7550, 1715, 1422
*R*_int_	0.061
(sin θ/λ)_max_ (Å^−1^)	0.626

Refinement	
*R*[*F* ^2^ > 2σ(*F* ^2^)], *wR*(*F* ^2^), *S*	0.030, 0.072, 0.91
No. of reflections	1715
No. of parameters	92
H-atom treatment	H-atom parameters not refined
Δρ_max_, Δρ_min_ (e Å^−3^)	0.36, −0.38
Absolute structure	Flack (1983 ▸)
Absolute structure parameter	0.02 (2)

Computer programs: *APEX2* and *SAINT* (Bruker, 2016 ▸), *SIR92* (Altomare *et al.*, 1994 ▸), *SHELXL2013* (Sheldrick, 2015 ▸), *ORTEP* for Windows and *WinGX* publication routines (Farrugia, 2012 ▸).

## full crystallographic data

### Crystal data

**Table:**

C_7_H_7_Br_2_N	*F*(000) = 504
*M**_r_* = 264.96	*D*_x_ = 2.105 Mg m^−^^3^
Orthorhombic, *P*2_1_2_1_2_1_	Mo *K*α radiation, λ = 0.71073 Å
Hall symbol: P 2ac 2ab	Cell parameters from 7750 reflections
*a* = 4.3773 (7) Å	θ = 2.1–26.4°
*b* = 13.585 (2) Å	µ = 9.62 mm^−^^1^
*c* = 14.057 (3) Å	*T* = 200 K
*V* = 835.9 (2) Å^3^	Needle, colorless
*Z* = 4	0.12 × 0.05 × 0.04 mm

### Data collection

**Table:**

Bruker APEXII QUAZAR CCD diffractometer	1715 independent reflections
Radiation source: ImuS	1422 reflections with *I* > 2σ(*I*)
Graphite monochromator	*R*_int_ = 0.061
Detector resolution: 8.02 pixels mm^-1^	θ_max_ = 26.4°, θ_min_ = 2.1°
f\ and ω scans	*h* = −5→5
Absorption correction: multi-scan (SADABS; Bruker, 2016)	*k* = −15→16
*T*_min_ = 0.396, *T*_max_ = 0.746	*l* = −17→17
7550 measured reflections	

### Refinement

**Table:**

Refinement on *F*^2^	Secondary atom site location: difference Fourier map
Least-squares matrix: full	Hydrogen site location: inferred from neighbouring sites
*R*[*F*^2^ > 2σ(*F*^2^)] = 0.030	H-atom parameters not refined
*wR*(*F*^2^) = 0.072	*w* = 1/[σ^2^(*F*_o_^2^) + (0.0409*P*)^2^] where *P* = (*F*_o_^2^ + 2*F*_c_^2^)/3
*S* = 0.91	(Δ/σ)_max_ = 0.001
1715 reflections	Δρ_max_ = 0.36 e Å^−^^3^
92 parameters	Δρ_min_ = −0.38 e Å^−^^3^
0 restraints	Absolute structure: Flack (1983)
0 constraints	Absolute structure parameter: 0.02 (2)
Primary atom site location: structure-invariant direct methods	

### Special details

**Table:**

Geometry. Bond distances, angles etc. have been calculated using the rounded fractional coordinates. All su's are estimated from the variances of the (full) variance-covariance matrix. The cell esds are taken into account in the estimation of distances, angles and torsion angles
Refinement. Refinement of F^2^ against ALL reflections. The weighted R-factor wR and goodness of fit S are based on F^2^, conventional R-factors R are based on F, with F set to zero for negative F^2^. The threshold expression of F^2^ > 2sigma(F^2^) is used only for calculating R-factors(gt) etc. and is not relevant to the choice of reflections for refinement. R-factors based on F^2^ are statistically about twice as large as those based on F, and R- factors based on ALL data will be even larger.

### Fractional atomic coordinates and isotropic or equivalent isotropic displacement parameters (Å^2^)

**Table:**

	*x*	*y*	*z*	*U*_iso_*/*U*_eq_	
Br1	0.69454 (12)	0.51851 (3)	0.40790 (4)	0.0449 (2)	
Br2	0.49325 (11)	0.11063 (3)	0.35184 (3)	0.0374 (2)	
N1	0.4343 (8)	0.3124 (3)	0.4488 (2)	0.0340 (14)	
C1	0.6072 (9)	0.3165 (3)	0.3674 (3)	0.0255 (14)	
C2	0.7481 (9)	0.4015 (3)	0.3360 (3)	0.0277 (14)	
C3	0.9295 (10)	0.4045 (3)	0.2553 (3)	0.0323 (17)	
C4	0.9781 (10)	0.3217 (3)	0.2004 (3)	0.0313 (14)	
C5	1.1658 (11)	0.3259 (3)	0.1108 (3)	0.0447 (17)	
C6	0.8409 (10)	0.2336 (3)	0.2315 (3)	0.0317 (14)	
C7	0.6636 (10)	0.2322 (3)	0.3118 (3)	0.0280 (12)	
H1	1.33755	0.28238	0.11654	0.0669*	
H1A	0.40957	0.36443	0.48284	0.0407*	
H1B	0.35064	0.25790	0.46576	0.0407*	
H2	1.02080	0.46361	0.23780	0.0388*	
H3	0.87095	0.17585	0.19722	0.0378*	
H4	1.23699	0.39194	0.10090	0.0669*	
H5	1.04240	0.30603	0.05765	0.0669*	

### Atomic displacement parameters (Å^2^)

**Table:**

	*U*^11^	*U*^22^	*U*^33^	*U*^12^	*U*^13^	*U*^23^
Br1	0.0576 (3)	0.0274 (2)	0.0497 (3)	−0.0005 (2)	0.0057 (3)	−0.0016 (2)
Br2	0.0373 (3)	0.0279 (2)	0.0471 (3)	−0.0053 (2)	−0.0027 (3)	0.0002 (2)
N1	0.038 (3)	0.031 (2)	0.033 (2)	−0.0013 (18)	0.0070 (18)	0.0005 (17)
C1	0.0174 (19)	0.028 (2)	0.031 (3)	0.0015 (17)	−0.0052 (19)	0.007 (2)
C2	0.023 (2)	0.027 (2)	0.033 (3)	0.0023 (18)	−0.0039 (19)	0.0002 (19)
C3	0.027 (3)	0.029 (3)	0.041 (3)	0.0011 (19)	0.001 (2)	0.006 (2)
C4	0.021 (2)	0.041 (3)	0.032 (2)	0.005 (2)	0.000 (2)	0.007 (2)
C5	0.036 (3)	0.057 (3)	0.041 (3)	0.009 (3)	0.005 (3)	0.008 (3)
C6	0.030 (2)	0.038 (3)	0.027 (2)	0.004 (2)	−0.004 (2)	−0.002 (2)
C7	0.024 (2)	0.028 (2)	0.032 (2)	0.001 (2)	−0.006 (2)	−0.0010 (19)

### Geometric parameters (Å, º)

**Table:**

Br1—C2	1.898 (4)	C4—C5	1.505 (6)
Br2—C7	1.898 (4)	C4—C6	1.409 (6)
N1—C1	1.373 (5)	C6—C7	1.370 (6)
N1—H1A	0.8600	C3—H2	0.9300
N1—H1B	0.8600	C5—H1	0.9600
C1—C7	1.408 (6)	C5—H4	0.9600
C1—C2	1.382 (6)	C5—H5	0.9600
C2—C3	1.385 (6)	C6—H3	0.9300
C3—C4	1.381 (6)		
			
C1—N1—H1B	120.00	Br2—C7—C1	118.3 (3)
H1A—N1—H1B	120.00	Br2—C7—C6	118.6 (3)
C1—N1—H1A	120.00	C1—C7—C6	123.0 (4)
C2—C1—C7	115.1 (4)	C2—C3—H2	119.00
N1—C1—C7	121.8 (4)	C4—C3—H2	119.00
N1—C1—C2	123.1 (4)	C4—C5—H1	110.00
Br1—C2—C1	118.3 (3)	C4—C5—H4	110.00
Br1—C2—C3	118.8 (3)	C4—C5—H5	109.00
C1—C2—C3	122.8 (4)	H1—C5—H4	109.00
C2—C3—C4	121.5 (4)	H1—C5—H5	109.00
C5—C4—C6	121.7 (4)	H4—C5—H5	109.00
C3—C4—C5	121.4 (4)	C4—C6—H3	120.00
C3—C4—C6	116.9 (4)	C7—C6—H3	120.00
C4—C6—C7	120.6 (4)		
			
N1—C1—C2—Br1	−1.0 (5)	Br1—C2—C3—C4	179.7 (3)
N1—C1—C2—C3	178.1 (4)	C1—C2—C3—C4	0.6 (7)
C7—C1—C2—Br1	−178.5 (3)	C2—C3—C4—C5	177.7 (4)
C7—C1—C2—C3	0.6 (6)	C2—C3—C4—C6	−1.5 (6)
N1—C1—C7—Br2	0.1 (6)	C3—C4—C6—C7	1.2 (6)
N1—C1—C7—C6	−178.5 (4)	C5—C4—C6—C7	−178.1 (4)
C2—C1—C7—Br2	177.6 (3)	C4—C6—C7—Br2	−178.5 (3)
C2—C1—C7—C6	−1.0 (6)	C4—C6—C7—C1	0.1 (7)

### Hydrogen-bond geometry (Å, º)

**Table:**

*D*—H···*A*	*D*—H	H···*A*	*D*···*A*	*D*—H···*A*
N1—H1*A*···Br1	0.86	2.65	3.077 (4)	112
N1—H1*B*···Br2	0.86	2.64	3.072 (4)	113
N1—H1*B*···N1^i^	0.86	2.38	3.120 (7)	144

Symmetry code: (i) *x*−1/2, −*y*+1/2, −*z*+1.
